# Intraoperative Use of a Topical Anesthetic Gel Versus Balanced Salt Solution During Cataract Surgery: Effects on Corneal Structure and Ocular Surface

**DOI:** 10.3390/jcm15051992

**Published:** 2026-03-05

**Authors:** Pier Giuseppe Ruggeri, Alberto Carnicci, Matilde Buzzi, Fabrizio Giansanti, Rita Mencucci

**Affiliations:** 1 Eye Clinic, Department of Neuroscience, Psychology, Pharmacology and Child Health (NEUROFARBA), University of Florence, Largo Brambilla 3, 50134 Florence, Italy; 2 Unit of Ophthalmology, Department of Medicine, Surgery and Neuroscience, University of Siena, Viale Mario Bracci 16, 53100 Siena, Italy

**Keywords:** cataract surgery, topical anesthesia, lidocaine gel, CCT, epithelial thickness, basal epithelial cell density, tear film stability, NI-BUT, Schirmer, OSDI

## Abstract

**Background/Objectives:** During cataract surgery, topical anesthesia is routinely achieved through the instillation of topical anesthetic eye drops, while different agents may be applied to the corneal surface during the procedure to support lubrication and protection. The impact of these intraoperative strategies on corneal integrity and postoperative ocular surface recovery remains an area of clinical interest. This study aimed to compare the intraoperative and postoperative effects of applying a topical anesthetic gel (Ophtesic, Horus Pharma) on the corneal surface versus the use of balanced salt solution (BSS) during cataract surgery. **Methods:** In this longitudinal, observational prospective study, 24 eyes of 24 patients undergoing phacoemulsification received either topical anesthetic gel (n = 15) or BSS irrigation (n = 9). Central corneal thickness (CCT) and epithelial thickness were measured preoperatively and on postoperative days 1, 5, and 15 using anterior segment optical coherence tomography (AS-OCT). Basal epithelial cell (BEC) density was assessed by in vivo confocal microscopy (IVCM), while OSDI score, non-invasive breakup time (NI-BUT), and Schirmer test I values were evaluated preoperatively and on postoperative days 5 and 15. Patient and surgeon satisfaction were rated using a Likert-like scale. **Results:** Both groups showed increased CCT and epithelial thickness at day 1. In the gel group, CCT returned to baseline by day 15 (*p* = 0.361), and epithelial thickness normalized by day 5 (*p* = 0.066). In the BSS group, CCT remained elevated at day 15 (*p* < 0.05), and epithelial thickness decreased at day 5 (*p* < 0.05) before returning to baseline. BEC density normalized at day 15 in the gel group (*p* = 0.107) but remained altered in the BSS group (*p* < 0.05). NI-BUT Schirmer I, and OSDI showed a trend toward faster recovery in the gel group than in the BSS group. **Conclusions:** In this exploratory study, intraoperative application of a topical anesthetic gel appeared to support early normalization of corneal and tear film parameters while providing effective anesthesia. Further studies are warranted to confirm these observations and evaluate potential long-term benefits.

## 1. Introduction

Cataract surgery is one of the most frequently performed ophthalmic procedures worldwide [1], and recent technological advances have significantly improved functional outcomes and patient satisfaction [2]. Despite these optimal results, many patients continue to experience foreign body sensation, burning, wind hypersensitivity, photophobia and reduced mechanical pain thresholds, all of which may substantially affect productivity and quality of life [2,3]. According to previous reports, postoperative pain is relatively common (34%) during the first hours after surgery [4]. Moreover, after hospital discharge, the prevalence of pain and ocular discomfort can persist at 24 h (10%), 1 week (9%) and even 6 weeks (7%) [4]. These symptoms are attributed to various mechanisms, such as corneal nerve damage, inflammation, goblet cell loss, phototoxicity from the operating microscope, worsening of meibomian gland dysfunction (MGD), use of eye drops before and after surgery and repeated intraoperative irrigation with balanced salt solution (BSS) [1,5,6,7,8].

Currently, topical anesthesia with anesthetic eye drops is considered the gold standard for cataract surgery. It is often preferred by high-volume surgeons and has been associated with fewer complications and lower costs compared with regional anesthetic techniques [9,10,11,12,13]. The advantages of topical anesthesia include a quicker visual recovery, a less invasive approach, preservation of ocular motility in cooperative patients, and avoidance of temporary cosmetic alterations [13]. However, topical anesthesia primarily affects superficial trigeminal fibers, leaving intraocular structures largely unanesthetized, which may result in increased discomfort during complex procedures requiring iris manipulation or posterior lens implant suturing. Assam et al. evaluated intraoperative pain during different steps of cataract surgery using topical proparacaine 0.5% and found significantly higher discomfort during hydrodissection and phacoemulsification compared with other surgical phases [14].

Recent evidence indicates that topical anesthesia with lidocaine 2% gel is a safe and effective alternative for clear-cornea cataract surgery. A potential advantage of gel anesthesia is its prolonged contact time with the ocular surface, which may enhance anesthetic efficacy [15]. Lidocaine 2% gel has been evaluated in clinical studies demonstrating effective intraoperative pain control [16]. Barequet et al. [17] reported that a single application of 0.25 inches of gel provided patients comfort comparable to three doses of tetracaine drops on a four-grade pain scale.

Moreover, several authors have introduced the use of corneal coating gels as an alternative to balanced salt solution irrigation to reduce the inflammatory and mechanical epithelial damage induced by intraoperative irrigation, while maintaining a clear optical reflex and minimizing corneal epithelial disruption [18].

In this context, an ophthalmic lidocaine hydrochloride gel 20 mg/g (Ophteesic, Horus Pharma) has been shown to provide long-lasting ocular surface hydration and effective epithelial protection when used as a corneal coating during cataract surgery [19]. This gel is transparent, uniform, and adheres well to the ocular surface; with a pH of 6.6—close to physiological values—and an osmolarity of 156 mOsmol/kg, it is gentle and comfortable on the cornea.

The aim of this prospective study was to compare the intraoperative and postoperative effects of applying a topical anesthetic gel for corneal protection versus the use of balanced salt solution during cataract surgery.

## 2. Materials and Methods

### 2.1. Study Design and Patients

This monocentric, observational, prospective study was conducted in accordance with the tenets of the Declaration of Helsinki and it was approved by the local Institutional Ethics committee. Verbal and written informed consent was obtained from all participants. Patient data were anonymized prior to access and analysis.

Patients undergoing standardized phacoemulsification surgery at the Eye Clinic of Careggi University Hospital in Florence, Italy, were enrolled between February and June 2025. Individuals with comparable clinical characteristics were included.

Exclusion criteria comprised dry eye disease (defined as Schirmer test < 10.0 mm, non-invasive breakup time [NI-BUT] < 10 s, or an ocular surface disease index [OSDI] score ≥ 23), previous corneal or other ocular surgery, contact lens wear, diabetes, glaucoma, corneal dystrophies, psychiatric or neurological disorders (including movement disorders and marked anxiety), eyelid abnormalities (such as entropion, ectropion and floppy eyelid syndrome), and the use of topical or systemic medications known to affect the ocular surface.

### 2.2. Surgical Technique and Postoperative Treatment

All procedures followed the standard surgical protocol of the Eye Clinic of Careggi. Three days before surgery, patients instilled bromfenac 0.9 mg/mL eye drops three times daily and performed eyelid hygiene twice daily. On the day of the surgery, mydriasis was achieved with phenylephrine/tropicamide drops (Visumidriatic Fenil 100 mg/mL + 5 mg/mL, Visufarma), instilled three times over 30 min preoperatively. Anesthetic eyedrops containing lidocaine 40 mg/mL were instilled topically before surgery in both groups.

Eyelid and conjunctival disinfection were performed using 10% and 5% povidone–iodine, respectively. Disinfection of the eyelid and conjunctival sac was done with 10% and 5% povidone iodine solution (Oftasteril, Alfa Intes srl, Naples, Italy) respectively.

In the gel coating group, according to the technical dossier of Ophtesic 20 mg/g ophthalmic gel [19], one gram of gel was applied to the corneal surface after disinfection and lid speculum insertion. No intraoperative BSS irrigation was used in this group, except at the end of the surgery, where 2 mL was used to gently rinse the ocular surface to remove residual gel.

In the BSS group, balanced salt solution was applied repeatedly during surgery, with a maximum volume of 15 mL.

All surgeries were performed by the same experienced surgeon (R.M.) using a standard temporal incision phacoemulsification technique (Centurion Vision System, Alcon Laboratories, Fort Worth, TX, USA) and posterior chamber foldable intraocular lens implantation. The cumulative dissipated energy (CDE) was recorded at the end of each procedure as automatically provided by the phacoemulsification system. Surgical time was calculated from the placement of the lid speculum to its removal at the end of surgery.

The postoperative regimen was the same in both groups and consisted of dexamethasone 1 mg/mL + netilmicin 3 mg/mL eye drops four times daily for two weeks, and bromfenac 0.9 mg/mL twice daily for one month.

### 2.3. Feedback Report

Patients remained under observation for at least 30 min after surgery. Before discharge, patient and surgeon satisfaction with anesthesia was evaluated using a Likert-like verbal rating scale. Participants responded to the question: “How do you evaluate your experience of the anesthesia during your operation?”, with possible scores from 1 (extremely dissatisfied) to 7 (extremely satisfied) [8,20].

Surgical duration (minutes) and any need for supplemental anesthesia or sedation were recorded.

### 2.4. Ocular Surface Examination

All patients underwent comprehensive ocular surface assessment. Eyelid abnormalities and the anterior segment were examined clinically and instrumentally.

Central corneal thickness (CCT) and epithelial thickness were measured using anterior segment optical coherence tomography (AS-OCT) with the MS-39 device (Costruzione Strumenti Oftalmici, Florence, Italy) by an experienced examiner (PGR). The instrument calculates epithelial thickness within the 8.0-mm zone and automatically generates an epithelial thickness map; epithelial thickness was specifically evaluated in the central 3.0-mm zone. Measurements were recorded preoperatively (3 days), and at postoperative day 1, day 5, and day 15.

Basal epithelial cell (BEC) density was evaluated using in vivo confocal microscopy (IVCM) with a field of view of 400 × 400 µm through Heidelberg Retina Tomograph II with Rostock Cornea Module (Heidelberg Engineering GmbH, Dossenheim, Germany) by the same experienced examiner (A.C.). BEC density was measured preoperatively (3 days) and at postoperative days 5 and 15.

Tear film evaluation included non-invasive breakup time (NI-BUT), Schirmer Test I, and the Ocular Surface Disease Index (OSDI) questionnaire. All measurements were performed preoperatively (3 days before surgery) and at 5 and 15 days postoperatively.

NI-BUT was measured using the lacrimal function module of the MS-39 device (Costruzione Strumenti Oftalmici, Florence, Italy). Patients were instructed to blink twice and then keep the eye open as long as possible; the time to first break in seconds was recorded.

Schirmer Test I was performed without anesthesia; patients closed their eyes gently for 5 min and the wetting length was measured thereafter [21].

### 2.5. Statistical Analysis

Statistical analysis was performed using IBM SPSS Statistics for Windows (version 30.0, IBM Corp.) and Stata software (version 19.0, StataCorp). Data distribution was assessed using the Shapiro–Wilks normality test. Means and standard error of mean (SEM) were calculated for all continuous parameters (age, CDE, surgery time, CCT, epithelial thickness, BEC density, NI-BUT, Schirmer I values and OSDI score).

Differences between preoperative and postoperative values for CCT, epithelial thickness, BEC density, TBUT, OSDI score, and Schirmer I values were evaluated using the Wilcoxon signed-rank test for paired samples. Comparisons between the coating gel and balanced salt solution groups for all parameters were performed using the Mann–Whitney U test for two independent samples. A *p*-value of less than 0.05 was considered statistically significant.

## 3. Results

Twenty-four eyes from twenty-four patients were enrolled. Fifteen patients were assigned to the Ophtesic Gel group and nine to the Balanced Salt Solution (BSS) observation group. The mean age was 72.8 ± 7.5 years in GEL group and 73.2 ± 3.6 years in BSS group. The two groups were comparable in terms of age, sex distribution, mean surgery time and CDE (Cumulative Dissipated Energy) (Table 1).

### 3.1. Feedback Report

With respect to the Likert-type verbal rating scale, both patients and the surgeon in the gel group reported high satisfaction scores (6.5/7 for patients; 6.8/7 for the surgeon), whereas scores were lower in the BSS group (4.2/7 for patients; 5/7 for the surgeon).

### 3.2. CCT and Epithelial Thickness

**Figure 1:**
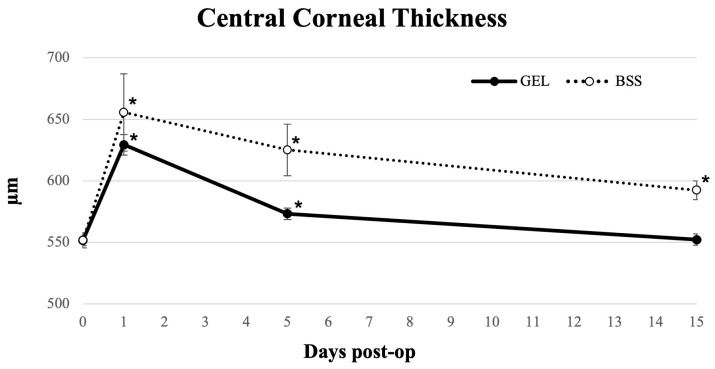
Longitudinal line graph describing central corneal thickness (CCT) in eyes undergoing cataract surgery treated with GEL versus BSS (* = statistically significantly different from baseline, *p* < 0.05).

CCT increased significantly in both groups of patients 1 day after the surgery (*p* < 0.05) and remained higher than the baseline at 5 days postoperatively (*p* < 0.05), as shown in Figure 1. In the coating gel group, CCT values at day 15 were comparable to baseline (*p* = 0.361), whereas in the BSS group, CCT remained significantly elevated (*p* < 0.05) (Table 2). Differences between groups were statistically significant at both day 5 and day 15 (*p* < 0.05).

**Figure 2:**
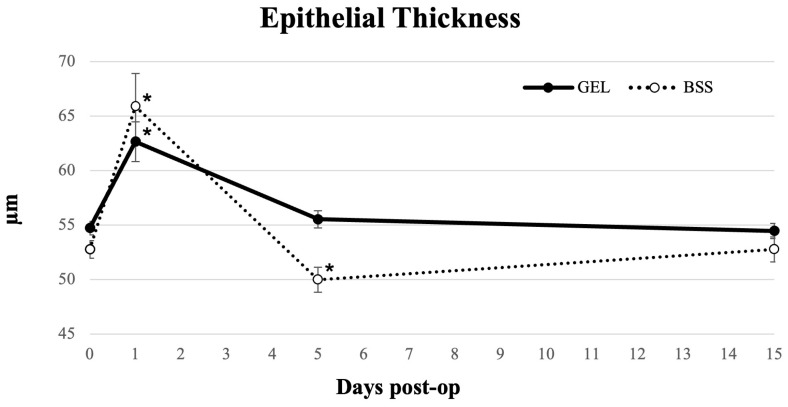
Longitudinal line graph describing epithelial thickness in eyes undergoing cataract surgery treated with GEL versus BSS (* = statistically significantly different from baseline, *p* < 0.05).

As shown in Figure 2, at 1 day postoperatively, the epithelial thickness increased significantly in all patients (*p* < 0.05). In the coating gel group, epithelial thickness returned to baseline by day 5 (*p* = 0.066) and showed no further significant changes (*p* = 0.478). In the BSS group, epithelial thickness decreased significantly at day 5 (*p* < 0.05) and returned to baseline by day 15 (*p* = 0.952) (Table 2). The epithelial thickness values were significantly lower in the patients treated with BSS than in those treated with the corneal coating gel at 5 days post-operatively (*p* < 0.05).

### 3.3. Basal Epithelial Cell Density

**Figure 3:**
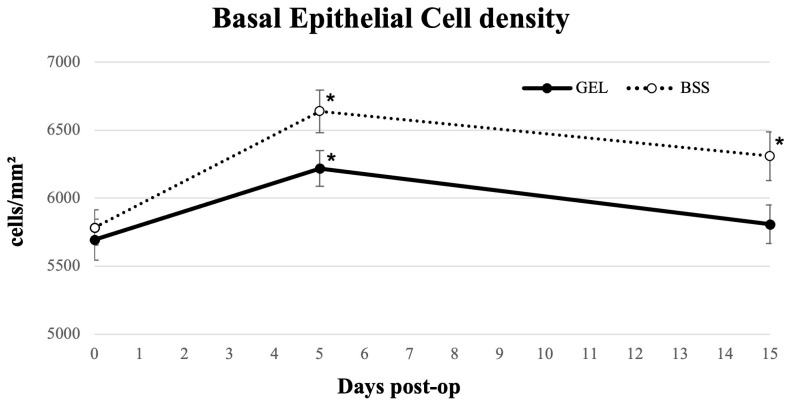
Longitudinal line graph describing basal epithelial cell density in eyes undergoing cataract surgery treated with GEL versus BSS (* = statistically significantly different from baseline, *p* < 0.05).

Figure 3 illustrates how, five days after surgery, BEC density increased significantly in both groups (*p* < 0.05). In the coating gel group, BEC density returned to baseline by day 15 (*p* = 0.107), whereas in the BSS group, the mean values were significantly different from the preoperative values at 15 days post-operative assessment (*p* < 0.05) (Table 2). The BEC density values were significantly higher at both 5 days and 15 days post-operatively in the BSS group than in the gel coating group (*p* < 0.05).

### 3.4. Tear Film Evaluation

**Figure 4:**
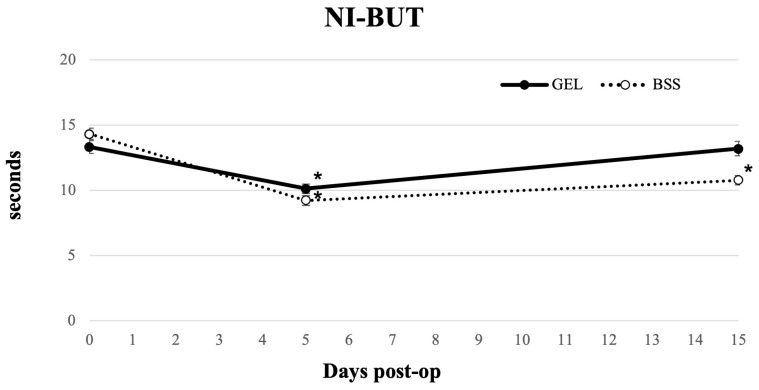
Longitudinal line graph describing Noninvasive break up time (NI-BUT) in eyes undergoing cataract surgery treated with GEL versus BSS (* = statistically significantly different from baseline, *p* < 0.05).

Regarding tear film stability (Figure 4), NI-BUT decreased at day 5 in both groups (*p* < 0.05). At 15 days postoperatively, the NI-BUT values returned to the preoperative level in the coating gel group (*p* = 0.484), while they remained significantly lower in the BSS group (*p* < 0.05) (Table 2). NI-BUT was significantly lower in the BSS group at day 15 compared with the gel group (*p* < 0.05).

**Figure 5:**
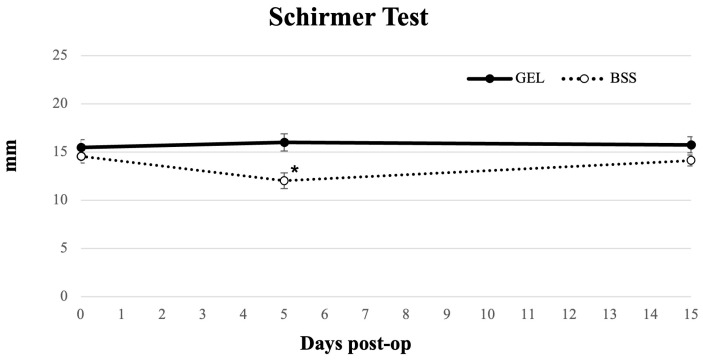
Longitudinal line graph describing Schirmer test results in eyes undergoing cataract surgery treated with GEL versus BSS (* = statistically significantly different from baseline, *p* < 0.05).

Tear production assessed by the Schirmer I test (Figure 5) returned to baseline by day 5 in the coating gel group (*p* = 0.492) and remained stable at day 15 (*p* = 0.182). In contrast, the BSS group showed significantly reduced tear production at day 5 (*p* < 0.05), with a return to baseline by day 15 (*p* = 0.407) (Table 2). Schirmer values were significantly higher in the gel group than in the BSS group at day 5 (*p* < 0.05).

**Figure 6:**
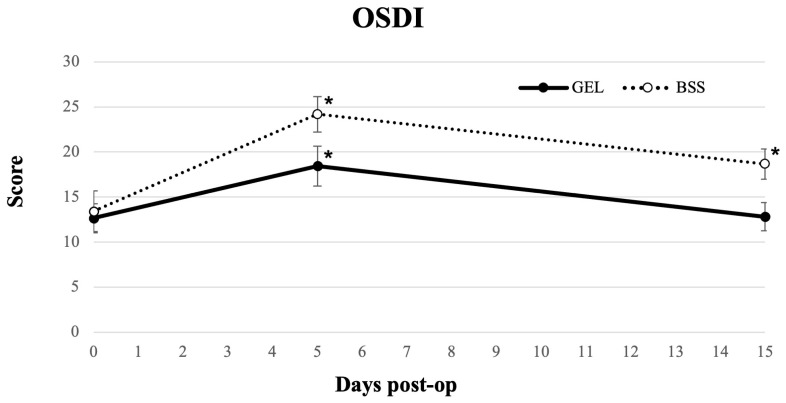
Longitudinal line graph describing Ocular Surface Disease Index (OSDI) test results in eyes undergoing cataract surgery treated with GEL versus BSS (* = statistically significantly different from baseline, *p* < 0.05).

The mean OSDI scores worsened significantly in both groups at day 5 compared with baseline (Figure 6). Scores returned to preoperative levels at day 15 in the coating gel group (*p* = 0.599) but remained significantly elevated in the BSS group (*p* < 0.05) (Table 2). Although OSDI values were consistently higher in the BSS group at both day 5 and day 15, these differences did not reach statistical significance (*p* = 0.147 and *p* = 0.056, respectively).

## 4. Discussion

Cataract surgery is currently among the most commonly performed ophthalmic procedures worldwide. The standard anesthetic approach relies on topical anesthetic eye drops, a method often preferred by high-volume surgeons due to its lower complication rate and reduced costs compared with regional anesthesia techniques [9,10,11,12]. However, cataract surgery is also recognized as a contributing factor to medically induced dry eye, leading to patient discomfort, visual disturbances, and suboptimal surgical outcomes [22]. Numerous studies have demonstrated a noticeable worsening of both signs and symptoms of dry eye following phacoemulsification, with reductions in tear film stability (BUT), decreased Schirmer I test values, and increased OSDI scores. These alterations may arise early and persist for up to three months postoperatively compared with baseline [6,23,24,25]

Previous studies suggest that corneal epithelial damage may result from prolonged exposure to microscopic surgical lights, increased inflammatory markers in the tear film due to ocular surface irritation, and direct surgical manipulation [23,26]. Additionally, intense intraoperative irrigation can reduce goblet cell density, leading to a subsequent decline in BUT [5].

To mitigate these issues, viscous substances have been employed during ocular surgery to protect delicate tissues from intraoperative trauma [27]. Intraoperative strategies aimed at maintaining corneal hydration and minimizing surface trauma have been investigated in different settings, with studies showing that viscous gels can improve surgical conditions, providing better optical clarity during cataract surgery [28,29]. Earlier studies reported that the application of a corneal anesthetic gel during cataract surgery significantly reduced the need for frequent balanced salt solution irrigation and decreased the incidence of postoperative dry eye, while also improving subjective symptoms and ocular surface parameters [30,31].

Similar methodological approaches have been previously applied to investigate intraoperative corneal coatings for ocular surface protection, allowing detailed assessment of corneal and tear film changes with optical coherence tomography and confocal microscopy [2]. In the present study, this framework was adapted to evaluate the effects of an anesthetic gel formulation, providing descriptive insights into postoperative ocular surface recovery and tear film stabilization. Specifically, an ophthalmic lidocaine hydrochloride gel 20 mg/g (Ophtesic, Horus Pharma) was used as a corneal coating. This anesthetic gel formulation enhances intraocular penetration of lidocaine, as demonstrated by Bardocci et al., likely due to its prolonged contact time on the ocular surface and a higher pH (6.6 for the gel versus 5.9 for the eyedrops), which increases the non-ionized fraction of lidocaine capable of crossing biological membranes [15].

When applied intraoperatively, the gel not only provides topical anesthesia but also hydrates and protects the corneal epithelium, reducing mechanical trauma from instruments, irrigation, ultrasound energy, and phacoemulsification. Intraoperative application of this gel minimizes, and in some cases eliminates, the need for irrigation relative to standard BSS [31,32].

Higher satisfaction scores were observed in the gel group for both patients and the surgeon, indicating a more favorable reported intraoperative experience compared with standard BSS irrigation. Notably, ideal anesthesia was achieved within 20–60 s after gel instillation, and the gel maintained its integrity on the ocular surface while remaining optically effective for the entire duration of surgery.

Advances in AS-OCT have enabled detailed evaluation of postoperative corneal changes, including CCT and epithelial thickness. In alignment with previous studies on post-surgical corneal and epithelial remodeling, we observed a significant increase in CCT on the first postoperative day in both groups, likely related to endothelial stress [33]. However, in the gel-treated group, CCT values returned to near-baseline values within 15 days, whereas in the BSS group, they remained elevated at the same time point. This behavior is may be consistent with the hypothesis that early corneal swelling could be influenced by differences in intraoperative surface management, potentially facilitating a faster restoration of physiologic hydration and transparency.

Regarding epithelial thickness, both groups showed a notable increase one day post-surgery, due to inflammation and swelling. The BSS group showed a significant decrease on days 5 and 15. These observations align with the biphasic epithelial remodeling model described in the literature: an early edema-mediated thickening is followed by a transient thinning or normalization of the epithelium, likely resulting from a combination of cellular turnover, shedding of edematous cells, and inflammatory modulation [27,34]. Conversely, in the gel-treated group, epithelial thickness returned to pre-operative values within five days and remained stable throughout the follow-up. The gel may help limit excessive epithelial disruption during surgery, potentially providing some protection against mechanical trauma from surgical instruments or irrigation, and possibly reducing the extent of postoperative epithelial fluctuations.

These observations were further supported by IVCM, which showed an early increase in basal epithelial cell density post-surgery. Current literature confirms that phacoemulsification induces transient microstructural alterations in the corneal epithelium and sub-basal nerve plexus, driven by mechanical, thermal, and inflammatory stimuli. Several studies demonstrated that tear-film inflammatory mediators increase after surgery and closely correlate with the epithelial microstructural alterations detected by IVCM [24]. Protective strategies that reduce intraoperative desiccation and friction, including surface-coating gels and OVD-based shielding, have been shown to limit these microstructural alterations and accelerate epithelial recovery [31]. In our cohort, epithelial morphology returned to levels similar to preoperative values within five days in the gel-treated group, which is consistent with the patterns observed in previous reports.

Several studies have shown that dry eye symptoms commonly emerge within the first week after phacoemulsification, with variable recovery times [22,26,35]. A recent meta-analysis similarly reported early postoperative deterioration in tear film parameters, noting that Schirmer I values often worsen in the short-to-medium term, while tear BUT shows a marked short-term reduction and only partial recovery, likely due to altered corneal sensory input and reduced blink rate after surgery [36]. In our cohort, NI-BUT decreased in both groups by day 5, reflecting an early destabilization of the tear film. However, by day 15, NI-BUT had returned to preoperative levels in the gel group. This pattern is consistent with previous reports suggesting that mechanical stress and frequent irrigation can affect postoperative tear film stability. Schirmer I test results showed a similar trend: tear production normalized by day 5 in the gel group, whereas the BSS group exhibited a reduction at day 5 and recovered to baseline at day 15. These findings align with previous observations, indicating that minimizing ocular surface desiccation and epithelial trauma may accelerate the restoration of aqueous tear production [31]. Subjective symptoms followed a similar pattern. Consistent with previous observations, OSDI scores worsened significantly at day 5 in both groups and showed a trend toward recovery by day 15 in the gel group, while remaining elevated in the BSS group [37]. However, between-group differences at both follow-up time points did not reach statistical significance, and these observations should be interpreted as descriptive trends rather than evidence of treatment superiority.

Several limitations of our study should be acknowledged. First, the sample size was relatively small, unbalanced between groups, and monocentric, which may limit the generalizability of the results and prevent robust statistical conclusions. Second, the follow-up period was short (15 days), preventing assessment of longer-term ocular surface recovery and potential late complications. Third, the study had an observational, non-randomized, and non-masked design, introducing the possibility of selection bias and precluding causal inference. Additionally, formal thresholds for defining clinically meaningful changes were not established, so the observed differences should be interpreted cautiously and considered as indicative trends rather than definitive evidence of clinical effect. Given these limitations, the results should be interpreted as exploratory associations.

Future randomized controlled trials with larger, balanced cohorts and extended follow-up are warranted to confirm these findings and to further investigate the long-term impact of corneal coating gels on ocular surface health and visual outcomes after cataract surgery.

## 5. Conclusions

In this prospective exploratory study, the intraoperative application of ophthalmic lidocaine hydrochloride gel 20 mg/g (Ophtesic, Horus Pharma) during phacoemulsification was associated with earlier normalization of selected corneal structural parameters and tear film indices compared with repeated BSS irrigation. In particular, trends toward faster recovery of CCT, epithelial thickness, and basal epithelial cell density were observed in the gel group, accompanied by a more rapid stabilization of tear film parameters. The use of the gel was also associated with higher patient and surgeon satisfaction, likely due to improved intraoperative comfort, reduced need for repeated irrigation, and better preservation of corneal clarity.

These findings suggest that the use of a topical anesthetic gel as a corneal coating may represent a potentially protective intraoperative strategy for the ocular surface. However, given the observational design, the present results should be interpreted with caution and cannot establish causal superiority.

Future adequately powered randomized controlled trials with longer follow-up are warranted to further assess the long-term impact of corneal coating gels on ocular surface integrity, nerve morphology, and visual quality after cataract surgery.

## Figures and Tables

**Table 1:** Demographic and intraoperative characteristics of included patients.

Parameter	GEL Group	BSS Group
**Number of patients (eyes)**	15 (15)	9 (9)
**Age (years)**		
*Mean ± SEM*	72.8 ± 1.9	73.2 ± 1.2
*Median (range)*	73 (60–85)	72 (65–89)
**Sex**		
*Male*	9	4
*Female*	6	5
**Surgery time (min)**		
*Mean ± SEM*	15.3 ± 1.4	17.4 ± 2.1
*Median (range)*	14 (9–27)	18 (10–26)
**CDE (%)**		
*Mean ± SEM*	13.57 ± 1.68	12.94 ± 2.53
*Median (range)*	13.62 (3.23–24.98)	11.46 (2.34–27.54)

**Table 2:** Corneal, epithelial, and tear film parameters measured at different postoperative follow-up time points in the GEL and BSS groups. Data are reported as mean ± SEM at baseline and at postoperative days 1, 5, and 15. *p* values indicate comparisons with baseline within each group (Wilcoxon signed-rank test).

Parameter	Pre-Op		Day 1 Post-Op		Day 5 Post-Op		Day 15 Post-Op	
	*Mean ± SEM*	*p Value*	*Mean ± SEM*	*p Value*	*Mean ± SEM*	*p Value*	*Mean ± SEM*	*p Value*
**CCT (µm)**								
*GEL group*	551.60 ± 4.34	N/A	629.27 ± 8.40	<0.05	573.27 ± 4.65	<0.05	552.13 ± 4.58	0.361
*BSS group*	551.67 ± 6.24	N/A	655.56 ± 31.53	<0.05	625.11 ± 20.96	<0.05	592.33 ± 7.68	<0.05
**Epithelial Thickness (µm)**								
*GEL group*	54.73 ± 0.62	N/A	62.67 ± 1.82	<0.05	55.53 ± 0.79	0.066	54.47 ± 0.70	0.478
*BSS group*	52.78 ± 0.80	N/A	65.89 ± 3.04	<0.05	50.00 ± 1.14	<0.05	52.78 ± 1.15	0.952
**BEC density (cells/mm^2^)**								
*GEL group*	5694 ± 150	N/A	-	N/A	6218 ± 131	<0.05	5809 ± 142	0.107
*BSS group*	5785 ± 130	N/A	-	N/A	6639 ± 156	<0.05	6309 ± 178	<0.05
**NI-BUT (sec)**								
*GEL group*	13.33 ± 0.49	N/A	-	N/A	10.13 ± 0.35	<0.05	13.20 ± 0.55	0.484
*BSS group*	14.33 ± 0.44	N/A	-	N/A	9.22 ± 0.36	<0.05	10.78 ± 0.36	<0.05
**Schirmer Test (mm)**								
*GEL group*	15.47 ± 0.81	N/A	-	N/A	16.00 ± 0.90	0.492	15.73 ± 0.84	0.182
*BSS group*	14.56 ± 0.71	N/A	-	N/A	12,00 ± 0.82	<0.05	14.11 ± 0.56	0.407
**OSDI (score)**								
*GEL group*	12.65 ± 1.60	N/A	-	N/A	18.44 ± 2.22	<0.05	12.82 ± 1.58	0.599
*BSS group*	13.42 ± 2.26	N/A	-	N/A	24.18 ± 1.95	<0.05	18.66 ± 1.68	<0.05

## Data Availability

The raw data supporting the conclusions of this article will be made available by the authors on request.
